# Axons of cortical basket cells originating from dendrites develop higher local complexity than axons emerging from basket cell somata

**DOI:** 10.1242/dev.202305

**Published:** 2023-11-20

**Authors:** Steffen Gonda, Christian Riedel, Andreas Reiner, Ina Köhler, Petra Wahle

**Affiliations:** ^1^ Developmental Neurobiology, Faculty of Biology and Biotechnology, Ruhr University Bochum, 44801 Bochum, Germany; ^2^ Cellular Neurobiology, Faculty of Biology and Biotechnology, Ruhr University Bochum, 44801 Bochum, Germany

**Keywords:** Axo-somatic, Axo-dendritic, Axon-carrying dendrites, Bitufted neurons, Activity-dependent growth, Organotypic cultures, Rodent neocortex

## Abstract

Neuronal differentiation is regulated by neuronal activity. Here, we analyzed dendritic and axonal growth of Basket cells (BCs) and non-Basket cells (non-BCs) using sparse transfection of channelrhodopsin-YFP and repetitive optogenetic stimulation in slice cultures of rat visual cortex. Neocortical interneurons often display axon-carrying dendrites (AcDs). We found that the AcDs of BCs and non-BCs were, on average, the most complex dendrites. Further, the AcD configuration had an influence on BC axonal development. Axons originating from an AcD formed denser arborizations with more terminal endings within the dendritic field of the parent cell. Intriguingly, this occurred already in unstimulated BCs, and complexity was not increased further by optogenetic stimulation. However, optogenetic stimulation exerted a growth-promoting effect on axons emerging from BC somata. The axons of non-BCs neither responded to the AcD configuration nor to the optogenetic stimulation. The results suggest that the formation of locally dense BC plexuses is regulated by spontaneous activity. Moreover, in the AcD configuration, the AcD and the axon it carries mutually support each other's growth.

## Teaser

**Summary:** The morphological speciality of axons emerging from dendrites has been shown to be important during development: basket cell axons emerging from dendrites grow more rapidly and form denser terminal fields.

## INTRODUCTION

The cortical interneuron types are classified by distinct axonal projection and termination patterns (Meyer and Ferres-Torres, 1984). The type-specific features are created by transcription factors (Favuzzi et al., 2019), and genetic alterations evoke morphofunctional abnormalities. For example, in fast-spiking Basket cells (BCs), mechanistic target of rapamycin complex 1 (mTORC1) haploinsufficiency accelerates the development of axon terminal elements, which is followed by a loss of perisomatic innervation in the adult mouse cortex, suggesting a role of mTOR for maintenance of parvalbumin cell connectivity (Amegandjin et al., 2021). Sox6 is required for parvalbumin bouton growth and stability in mouse cortex (Munguba et al., 2021). Lack of MeCP2 accelerates development of parvalbumin cells in mouse cortex *in vivo* as well as in organotypic culture (OTC) (Patrizi et al., 2020). Yet, genetic determination does not shield interneurons from environmental influences, in particular neuronal activity. For mouse visual cortical interneurons, ablating the retinal input causes a loss of dendritic spines and synapses (Keck et al., 2011). Overexpression of GluA1(Q)flip and GluK2 glutamate receptor subunits increases dendritic complexity and spine density of rat visual cortical interneurons (Hamad et al., 2011; Jack et al., 2019). Axons of rat visual cortical interneurons silenced via activation of hM4Di designer receptor display the same degree of complexity and bouton size as mock-stimulated neurons, but have less bouton terminaux, a proxy of presynapses (Gasterstädt et al., 2022). Silenced mouse cortical BCs retain a normal complexity but innervate a lower number of pyramidal cells presumably due to enhanced pruning of terminal elements (Chattopadhyaya et al., 2004; Di Cristo et al., 2004; Baho and Di Cristo, 2012). Reducing the GABA production and the amount of GABA released from mouse cortical BCs impairs their ability to stabilize axo-somatic synapses (Chattopadhyaya et al., 2007). In BCs of rat visual cortex, inflammatory cytokine-evoked hyperexcitability lowers GAD, Kv3.2 and synaptotagmin 2 expression, evokes calcium events at higher frequencies but lower amplitude, and while the axonal complexity remains unchanged, the number and the size of axonal boutons declines (Engelhardt et al., 2018). Further, axonal geometry of postnatal day (P) 12-P18 mouse hippocampal chandelier cells is shaped through remodeling (Steinecke et al., 2017), and axo-axonic boutons decline in number when activity increases because the chandelier cell action at the axon initial segment is depolarizing at this age (Pan-Vasquez et al., 2020). Of the non-fast-spiking non-Basket cells (non-BCs) of mouse cortex, reducing excitability via expression of Kir2.1 channels from late fetal to P8 reduces axonal complexity of calretinin and reelin neurons derived from the caudal ganglionic eminence (De Marco García et al., 2011).

The ability to reorganize BC axons persists in the adult. Within hours of sensory deprivation by whisker plucking in mice, the horizontal axons of deprived barrel cortex BCs discard local collaterals and sprout into non-deprived barrels almost doubling their length (Marik et al., 2010). Mouse prelimbic parvalbumin cells grow more complex axons upon chemogenetic stimulation (Stedehouder et al., 2018). Further, epilepsy has been found to decrease mouse dentate gyrus axo-axonic synapse density while the density of axo-somatic synapses increases (Alhourani et al., 2020).

Together, the evidence supports the view that the morphological development of interneuronal axons is regulated by activity. The activity-dependent regulation of terminal elements and boutons has been well characterized in slice cultures at week 3-4, which revealed the importance of GABA synthesis for terminal element development (Chattopadhyaya et al., 2004, 2007; Di Cristo et al., 2004; Baho and Di Cristo, 2012). However, the potential impact of cell activity on the total dimensions of the axon plexus was hardly ever assessed. Interneuronal axons frequently emerge from dendrites (Wahle et al., 2022). Axon-carrying dendrites (AcDs) of mouse hippocampal pyramidal neurons have been reported to be privileged with a higher excitability and the ability to elicit action potential firing, which escapes somatic inhibition; also the pyramidal neuron AcD is longer than regular dendrites (Thome et al., 2014; Hodapp et al., 2022). Yet, it is unknown whether the AcD configuration has a morphogenetic impact on interneuronal dendrites or axons. Here, we report that BC axons, but not non-BC axons, originating from an AcD formed locally denser arborizations with more terminal endings even without optogenetic stimulation. In contrast, channelrhodopsin-mediated optogenetic activation increased the local axonal density of BC axons of somatic origin. These results suggest that the development of local BC plexuses is promoted by spontaneous activity and that the AcD configuration has a morphogenetic role for developing dendrites and axons.

## RESULTS

### Cell type-specific effects of activity on dendritic complexity

**Fig. 1.**
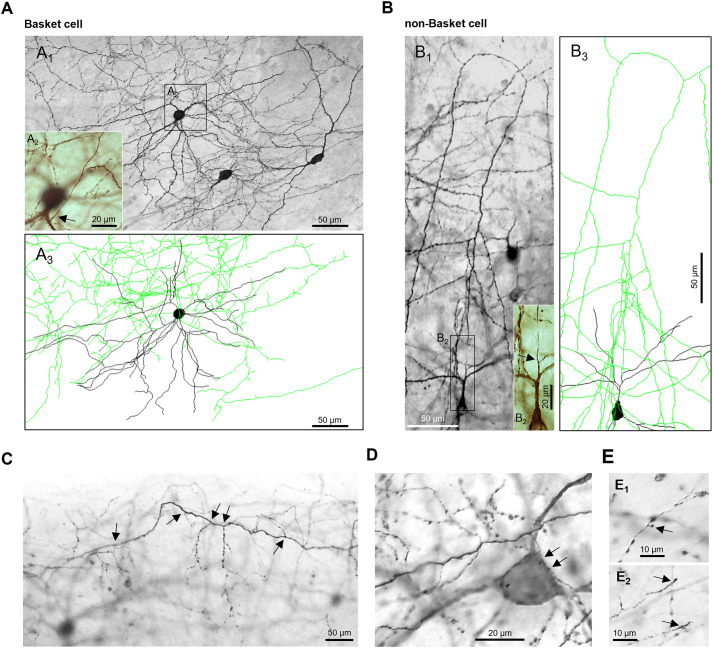
**Representative interneurons with AcDs.** (A) Basket cell; montage of eight photomicrographs showing cell overview (A1), the axon emerged from a dendrite close to the soma (A2) and the local arborization, cropped from the Neurolucida reconstruction of the neuron (A3). (B) Neuron with an arcade axon; montage of three photomicrographs showing the initial axonal branching (B1), the axon emerged from a second-order dendrite (B2) and the local arborization, cropped from the Neurolucida reconstruction of the neuron (B3). Immunohistochemical staining for transfected ChR2-YFP, diaminobenzidine reaction product intensified with osmium tetroxide for manual Neurolucida reconstruction at 1000× magnification. Axons in green, dendrites in black, somata in gray. Arrows indicate the axon hillock. (C) Photomicrograph of a horizontal axon of a BC with local branches and termina elements; branch points indicated by arrows; the parent soma is in the background at the lower left corner. (D) Thick main axon of another BC giving rise to a terminal branch contacting a pyramidal-shaped soma (arrows). (E) Axon collateral with a bouton terminaux (E1) and collaterals with growth cones at the tips (E2).

Roller tube cultures are spontaneously active, preserve the morphofunctional properties of the interneuron types and allow reconstruction of complete axons (Klostermann and Wahle, 1999; Di Cristo et al., 2004; Chattopadhyaya et al., 2007; Baho and Di Cristo, 2012; Feldmeyer et al., 2018). We reconstructed axons of ChR2-eYFP labeled BCs (Fig. 1A) and non-BCs with arcade (Fig. 1B) and bitufted morphology relying on classical axonal features (Meyer and Ferres-Torres, 1984; Klostermann and Wahle, 1999; Staiger et al., 2015; Markram et al., 2015; Feldmeyer et al., 2018) (Fig. 1; Fig. S1A-C). Inclusion criteria are in the Materials and Methods. In rodent cortex *in vivo*, the cell types become recognizable at ∼P10. Analysis of BC axon arborization in developing mouse cortex found that many presynapses are already close to their somatic targets at P14 (Micheva et al., 2021). Further, miniature inhibitory postsynaptic current (IPSC) frequency increases from P6-P15 in mouse somatosensory cortex pyramidal cells, with most IPSC originating from perisomatic synapses (Kobayashi et al., 2008; Micheva et al., 2021). Indeed, neurons at days *in vitro* (DIV) 15 in OTC were well differentiated, with BCs forming thick main axons giving rise to thin side branches with irregular-sized boutons (Fig. 1C), terminal elements contacting somata (Fig. 1D), bouton terminaux which are bona fide presynapses (Fig. 1E_1_). Growth cones suggested that axons of BCs and non-BCs were actively growing at DIV 15 (Fig. 1E_2_). Accordingly, optogenetic stimulation was applied from DIV 11-15, which overlaps the main growth phase of BCs.

**Fig. 2.**
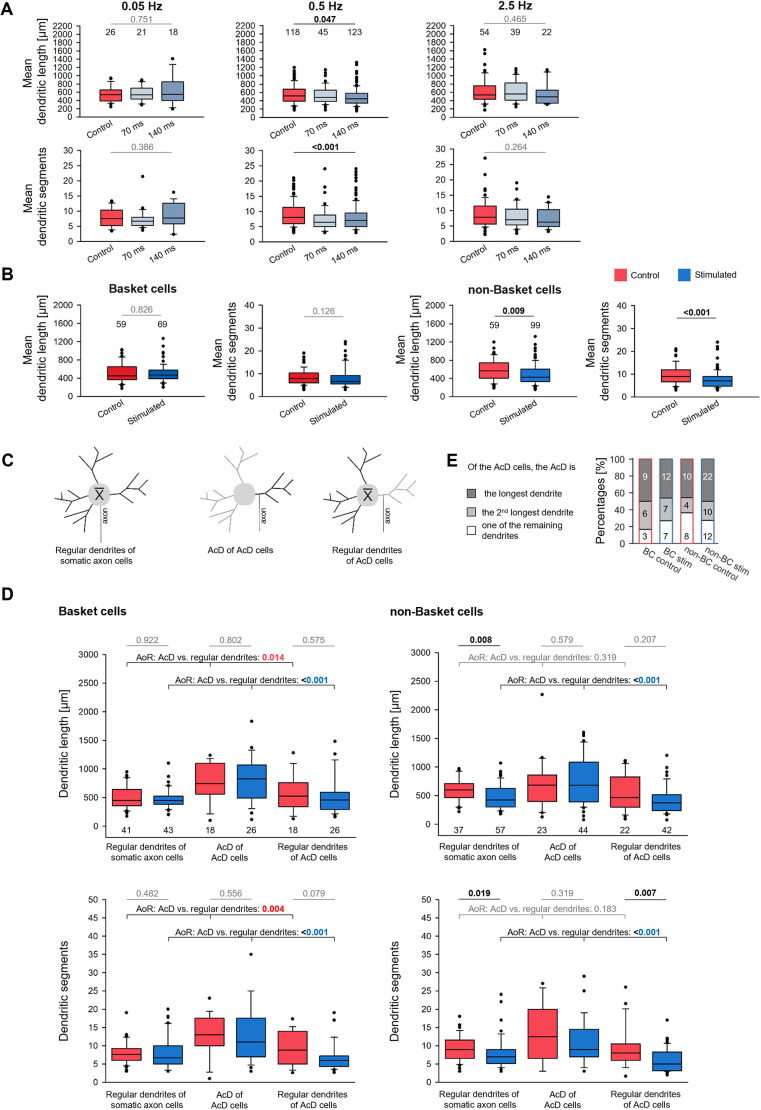
**Dendrites of interneurons.** (A) Box plots of mean dendritic length (top row) and mean dendritic segment numbers (bottom row) of all interneurons after DIV 11-15 ChR2-eYFP stimulation with 0.05 Hz, 0.5 Hz and 2.5 Hz, each with pulse duration of 70 ms and 140 ms (in shades of blue). The number above each box is the number of neurons analyzed. *P*-values determined with ANOVA on ranks corrected for multiple testing versus the handling control (in red). (B) Box plots of mean dendritic length (left) and mean dendritic segment numbers (right) of BCs and non-BCs. Handling control in red, stimulation with 70 ms and 140 ms conditions pooled in blue. The number above the boxes is the number of neurons analyzed. *P*-values determined with Mann–Whitney rank sum test versus the handling control. (C) Sketches depicting the measured dendrites (black). The AcD of AcD cells (middle) was compared with the average of all other dendrites of the AcD cell (right), and with the average of the dendrites of non-AcD cells (left). (D) Length (top row) and segments (bottom row) of the three groups of dendrites for BCs (left column) and non-BCs (right column). The number of cells is given below every box. The number of somatic axon cells is higher than the number of AcD cells. A total of 128 BC and 158 non-BC was analyzed. Of the latter, three cells had two axons from two dendrites, and these AcDs were included. First, we compared control with stimulated cells; Mann–Whitney rank sum test; *P*-values are the numbers above the lines. Second, we compared the AcD versus the regular dendrites (control cells in red, 0.5 Hz stimulated cells in blue); *P*-values determined with ANOVA on ranks (AoR). Significant differences in black or color, all other *P*-values in gray lettering. (E) Percentage of BCs and non-BCs in which the AcD is the longest, second longest or one of the remaining dendrites. Box plots show median (middle bars) and first to third interquartile ranges (boxes); whiskers indicate the 10th and 90th percentile range; dots indicate outliers.

Given the wealth of evidence that activity is promoting dendritic growth, we expected to see an increase of dendritic complexity with either one of the three selected light pulse frequencies or either one of the two light pulse durations. Only the 0.5 Hz stimulation at 70 ms and 140 ms pulse duration altered the interneuronal dendrites but, unexpectedly, dendrites were on average shorter and less branched compared with the handling controls (Fig. 2A; Table S1). The moderate decrease observed with the 0.5 Hz stimulation suggested that only one of the interneuron types was affected. As pulse duration appeared to be less important than pulse frequency, as seen in our previous study (Gonda et al., 2023), we pooled cells of the 70 ms and 140 ms condition for all following analyses. When analyzing BCs (pooled) and non-BCs (pooled) separately, we found that the 0.5 Hz stimulation evokes shorter and less branched dendrites specifically in non-BCs (Fig. 2B; Table S1).

The finding mirrored our recent observation of stunted apical dendritic growth of pyramidal neurons assessed from the very same set of OTCs. Also here, the effect has been only observed at 0.5 Hz (Gonda et al., 2023). The 0.05 Hz stimulation was not effective, and the 2.5 Hz stimulation elicited dendritic injury and cell death. We have suggested (Gonda et al., 2023) that the 0.05 Hz failed because it resembles the activity level typical for perinatal neurons, whereas calcium event frequencies of 2.5 Hz do not occur in visual cortex *in vivo* until well after P20 (Rochefort et al., 2009). This might explain why pyramidal cells and interneurons during the second postnatal week respond with morphological changes only to 0.5 Hz, which comes closest to a frequency range the neurons are going to reach in their next developmental phase.

Substantial numbers of axons emerge from dendrites, termed AcD cells and AcDs, respectively. In mouse, hippocampal AcD pyramidal cells are preferentially recruited during ripple oscillations, and the AcDs display a higher excitability and an ability to elicit action potential firing, which escapes somatic inhibition (Thome et al., 2014; Hodapp et al., 2022). Further, the basal AcD of CA1 pyramidal cells of 5- to 10-week-old mice are longer than regular dendrites and comprise about one-third of total basal dendritic length (Hodapp et al., 2022). However, thick-tufted mouse cortical layer V pyramidal neurons with a basal AcD display a reduced dendritic complexity and thinner main apical dendrite than those with somatic axons (Hamada et al., 2016). We therefore analyzed the AcD versus the average of the regular dendrites as depicted in Fig. 2C (see also Table S1). For BCs, AcDs of both the control cells and the 0.5 Hz stimulated cells were significantly longer and more branched than the average dendrite of somatic axon cells (left column in Fig. 2D). The AcDs were not extremely long; instead, they remained within the range of the longest dendrites of all individual BCs of our sample. We did not have the impression that AcD interneurons were clustered. Any given OTC can have 1, 2 or no AcD interneuron, and the more transfectants the higher the chance to find an AcD interneuron.

For the non-BCs of the control condition (right column in Fig. 2D; Table S1), the AcD were within the range of the regular dendrites. The stimulated cells with somatic axons presented on average shorter and less branched dendrites as described above (Fig. 2B). The regular dendrites of the stimulated AcD cells had the lowest number of segments (right column in Fig. 2D; Table S1). Analyzing the AcD cells separately confirmed that the AcD represented the longest or second-longest dendrite in 60-80% of the cells (Fig. 2E). Together, this suggested that in BCs, the AcD configuration awards a morphogenetic advantage for the dendrite which carries the axon. Further, the AcD of stimulated non-BC interneurons remained as long as the AcD of control cells. This suggested that the AcD configuration awards resistance to the growth-impairing effect of the optogenetic stimulation which stunted the regular dendrites of the AcD cells. Also, stimulated pyramidal cells reconstructed from the same OTCs displayed shorter apical dendrites, and infragranular cells were more susceptible than supragranular cells (Gonda et al., 2023).

### Cell type-specific effects on axonal complexity

**Fig. 3.**
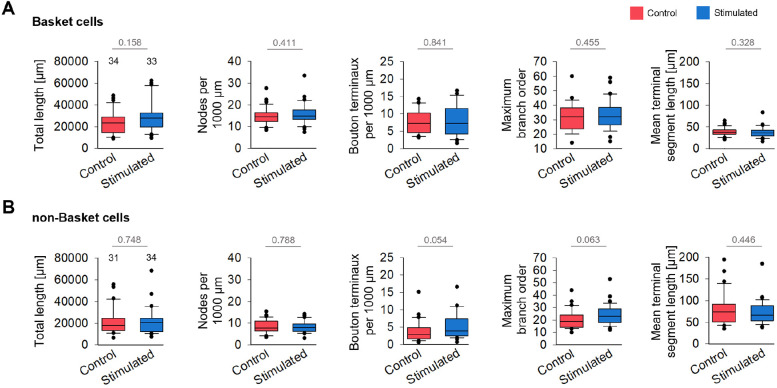
**Dimensions of BC and non-BC axons.** (A) For BC axons, total length, number of nodes, number of bouton terminaux, maximum branch order and terminal segment length were not influenced by the 0.5 Hz stimulation. Mann–Whitney rank sum test; *P*-values are the numbers above the lines. (B) For non-BC axons, total length, number of nodes, number of bouton terminaux, maximum branch order and terminal segment length were not influenced by the 0.5 Hz stimulation. Mann–Whitney rank sum test; *P*-values are the numbers above the lines. The number above the boxes is the number of neurons analyzed. Box plots show median (middle bars) and first to third interquartile ranges (boxes); whiskers indicate the 10th and 90th percentile range; dots indicate outliers.

We reconstructed the axons of BCs and non-BCs (70 ms and 140 ms conditions pooled). For BCs and non-BCs (Fig. 3A,B; Table S2) the total axonal length, the number of branch points of collaterals (nodes) per 1000 µm, the maximum branch order and the mean length of the terminal segments were not different from the handling control. Only the number of bouton terminaux tended to be higher in the stimulated non-BC axons. Comparing the two cell classes revealed differences which were to be expected. For example, BC axons were on average longer than axons of non-BCs, and BC axons had higher numbers of nodes, more bouton terminaux and a higher maximum branch order. Quite typically, the terminal segments of the BC axon collaterals were substantially shorter and far more uniform in length than the terminal segments of non-BC axon collaterals. BC axons of comparable dimensions have been reported for P19-P21 mouse dentate gyrus (Doischer et al., 2008). For non-BCs, reported values in mouse cortex at P21 (Lim et al., 2018) were slightly higher than our values, which might be ascribed to their slightly older age. Our axons at DIV 15 were still actively growing, yet our BC and non-BC axon dimension matched well with the dataset obtained from P19 rat frontal cortex (Karube et al., 2004). Therefore, interneuronal axons in OTC are at dimensions typical for their cell type and developmental stage.

**Fig. 4.**
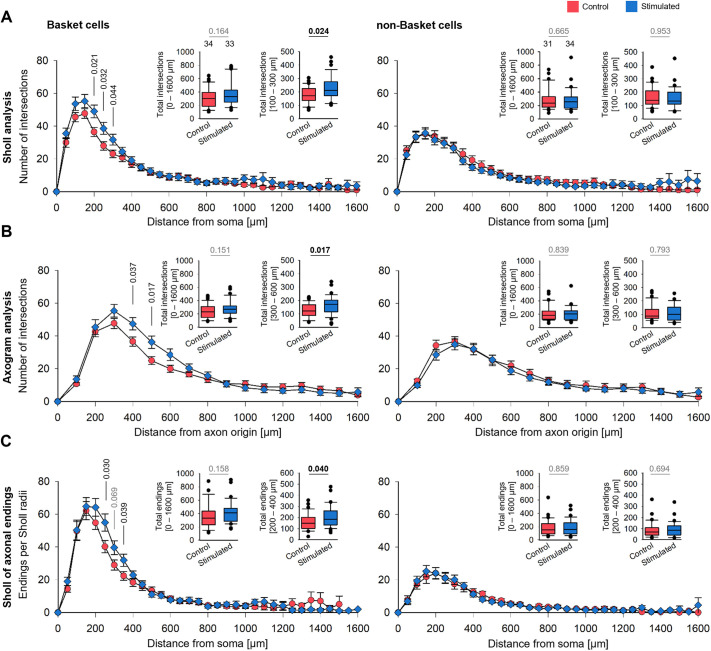
**Sholl-type analyses reveal denser arborizations of optogenetically stimulated BC, but not non-BC, axons.** (A) Soma-centered Sholl analysis. (B) Axogram analysis. (C) Collateral endings per soma-centered Sholl radii. The insets depict the total intersections at selected distances from the soma or from the axon origin, respectively (plotted as mean±s.e.m.). Note that the bins with significant effects are shifted in the axogram analysis as it is based on the linear distance from the start point, whereas the soma-centered Sholl analysis scores line crossings of collaterals weaving back and forth. *P*-values determined by Mann–Whitney rank sum tests. Significant differences in black, all other *P*-values in gray lettering. Handling control in red, stimulated axons in blue. Box plots show median (middle bars) and first to third interquartile ranges (boxes); whiskers indicate the 10th and 90th percentile range; dots indicate outliers.

Next, Sholl-type analyses were performed (Fig. 4; Fig. S2A,B; Table S3). A soma-centered Sholl analysis revealed that the 0.5 Hz stimulated BCs had overall more axonal intersections and significantly more intersections within the 100-300 µm radius from the soma (Fig. 4A, left). The difference became even clearer with the axogram analysis. Again, the stimulated BCs had significantly more axonal intersections at 300-600 µm linear distance from the axon origin (Fig. 4B, left). In line with this, BC axons had significantly more terminal endings within the 200-400 µm bins (Fig. 4C, left). No differences were seen for the non-BC axons in total intersection (Fig. 4A, right), with the axogram analysis (Fig. 4B, right) or with the terminal endings (Fig. 4C, right). As the non-BC axons were reconstructed from same set of OTCs, often enough from the very same culture, they represent a perfect internal control population that responded with dendritic but not axonal changes. Together, the analysis suggests that optogenetically stimulated BC axons form denser plexuses within and around the dendritic field of the parent soma.

**Fig. 5.**
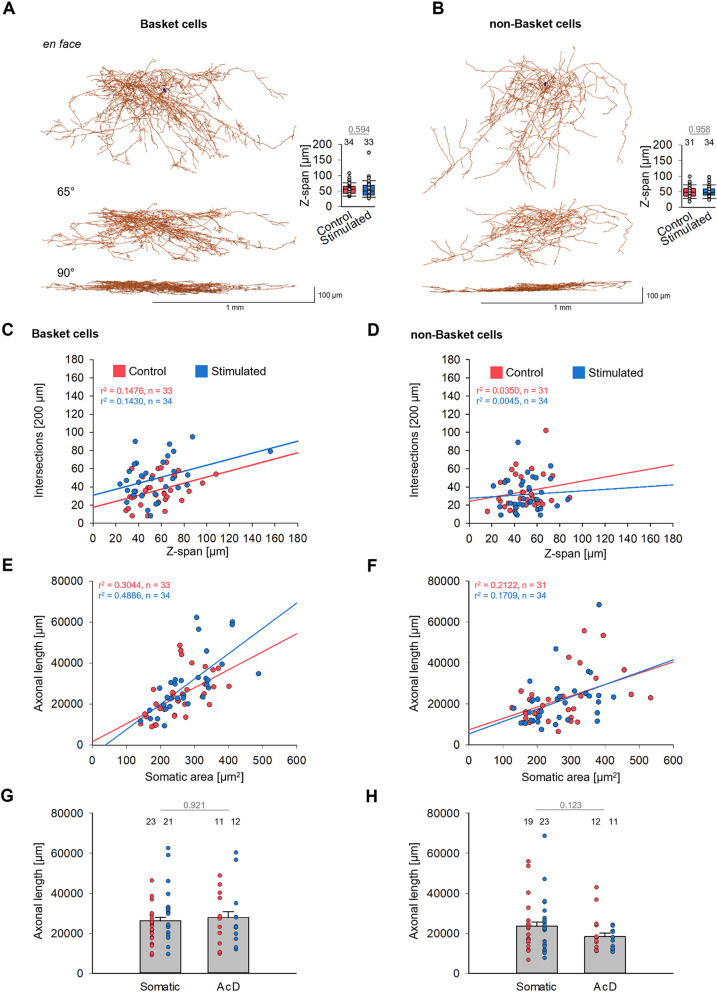
**Axonal dimensions do not depend on culture *z*-span and soma size.** (A,B) Representative BC (A) and non-BC (B) axon plexuses shown *en face* and rotated; somata in black. Insets show that control and 0.5 Hz stimulated BCs and non-BCs had on average the same *z*-span within the slice cultures. The number above the boxes is the number of neurons analyzed. *P*-values determined with Mann–Whitney rank sum test. Box plots show median (middle bars) and first to third interquartile ranges (boxes); whiskers indicate the 10th and 90th percentile range; dots indicate outliers. (C,D) Plots of Sholl circle intersections at 200 µm distance from the soma versus the *z*-span for BCs (C) and non-BCs (D). Every dot represents one neuron. (E,F) Plots of axon length versus soma size for BCs (E) and non-BCs (F) reveal somewhat stronger correlation, in particular for the BCs. Every dot is one neuron. The regression lines were computed using SigmaPlot12. (G,H) Length of somatic and AcD axons (gray bars; mean±s.e.m.), and upon separation by axonal origin (red and blue dots; every dot is one cell), for BCs (G) and non-BCs (H). The numbers above the symbols are the number of axons analyzed.

The lack of effect for non-BC axons already argued for a BC-specific effect. However, roller cultures flatten over time in an individual manner. Therefore, we had to rule out that variable degrees of flattening of individual OTC, or portions of them, resulted in sampling errors that might have led to the statistical differences seen for BC axons (Fig. 5; Table S4). Any difference in culture thickness should have affected both BC axons and non-BC axons. We thus analyzed the *z*-span for every cell after a 90° rotation (Fig. 5A,B), but no difference in *z*-spans was observed. Also, plotting the intersections at the (arbitrarily selected) 200 µm radius of the soma-centered Sholl versus the *z*-span yielded no conspicuous correlations as indicated by the low r^2^ values, neither for BC (Fig. 5C) nor non-BC (Fig. 5D) axons. As expected, the 0.5 Hz stimulated BC axons had shifted slightly upwards, indicating a higher number of intersections per unit *z*-span (Fig. 5C). This supported our finding of a stimulus-evoked increase in complexity specifically of the BC axon plexuses.

Interneurons differ in soma size, and large somata often support long axons. Analyzing axon length versus somatic area yielded a positive correlation for the stimulated BC cells (Fig. 5E) but not for non-BC cells (Fig. 5F). However, there may be no causal dependence, and we expected a certain correlation because it is well known that soma size itself is regulated by activity. Further, the axonal length was not correlated with axon origin (Fig. 5G,H). Finally, for a set of AcD cells, the distance between soma and axon origin was determined. The distance for BCs ranged from 4.9 µm to 18.8 µm (mean 9.8 µm; *n*=13 arbitrarily selected BCs) and for non-BCs the distance ranged from 1.8 µm to 32.4 µm (mean 12.2 µm; *n*=34).

### A morphogenetic role for the AcD of BCs

**Fig. 6.**
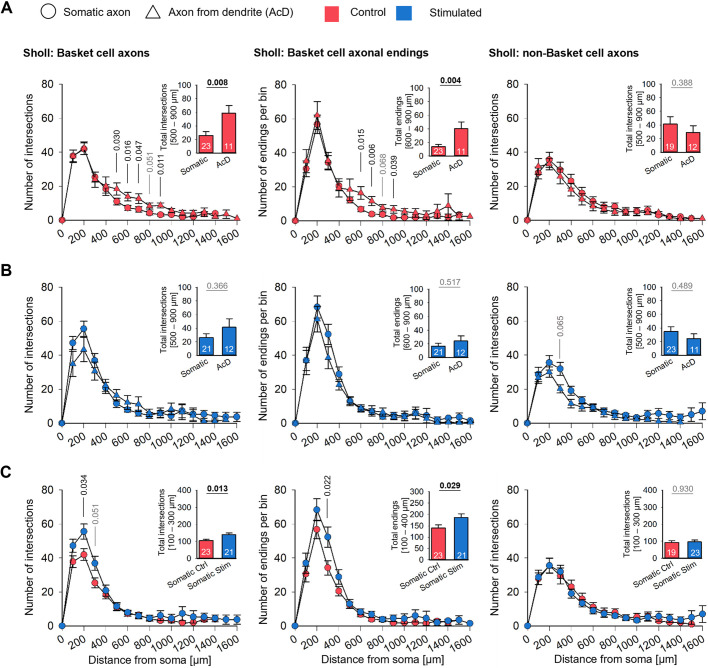
**Sholl-type analyses reveal a higher local complexity of BC axons originating from a dendrite.** (A) Comparison of control axons originating from somata or from dendrites. (B) Comparison of 0.5 Hz stimulated axons originating from somata or from dendrites. (C) Comparison of control and stimulated axons originating from somata. Left column, intersections of BC axons; middle column, axonal endings of BC axons; right column, intersections of non-BC axons. The insets show the total intersections in the selected bin (mean±s.e.m). Numbers in the bars are the number of axons analyzed. *P*-values determined by Mann–Whitney rank sum tests. Significant differences in black, all other *P*-values in gray lettering.

The AcD has been identified as belonging to the larger dendrites of BCs and non-BCs (Fig. 2D,E). When axons arise from a dendrite the two neurites share a joint segment. At the molecular level, dendrites and axons are characterized by different structural components. Competition for building material and transportation chains through the needle eye of the joint segment might put one of the neurites at a disadvantage. Thus, the axon from a dendrite might be shorter or less branched compared with an axon of somatic origin. On the other hand, the AcD is a dendrite with higher excitability, as determined for CA1 pyramidal cells (Thome et al., 2014; Hodapp et al., 2022). This may promote axonal development. We therefore reanalyzed the BC and non-BC sample, separating axons with AcD origin and with somatic origin (Fig. 6A-C; Table S5).

Of the control BCs, axons originating from a dendrite had significantly more intersections within 500-900 µm distance from the soma than axons originating from somata (Fig. 6A, left). Accordingly, the number of terminals was higher (Fig. 6A, middle). No such difference was seen for non-BC axons (Fig. 6A, right). Comparing intersections and terminal endings of the optogenetically stimulated BCs revealed that axons originating from somata were locally as dense as axons originating from an AcD (Fig. 6B, left and middle). For stimulated non-BC axons, the curves were not different (Fig. 6B, right). The effect became clear when comparing axons with somatic origin of control BCs to those of stimulated BCs: The stimulated axons had significantly more intersections within 100-300 µm distance to the soma (Fig. 6C, left) and more terminals within 100-400 µm distance to the soma (Fig. 6C, middle). Again, non-BC axons originating from an AcD were not different from somatic axons, and the optogenetic stimulation did not increase the local complexity (Fig. 6C, right).

Together, the results suggest that BC axons originating from a dendrite form denser local arborizations with more terminal endings within and around the dendritic field of their parent somata in the absence of an optogenetic stimulation. Thus, morphogenesis has been driven by spontaneous activity. Optogenetic stimulation then enabled BCs with somatic axons to reach the same degree of local arborization within the time given.

## DISCUSSION

The emergence of axons from dendrites of mammalian cortical neurons has been known for decades. Also, for decades, the functional implications have not been recognized. Recent work demonstrates that this neuromorphological feature is neither rare nor random. Rather, it segregates with species and cell types in the neocortex (Wahle et al., 2022). About 25% of the BCs and up to 50% of the non-BCs in cortex *in vivo* and in slice cultures have an axon originating from a dendrite (Höfflin et al., 2017; Wahle et al., 2022). This naturally occurring feature now delivers strong support for the view that the growth of BC dendrites and complexity of BC axons is driven by spontaneous activity. In line with our finding, the AcDs of hippocampal pyramidal cells are longer than regular dendrites of cells with somatic axons and, further, the hippocampal AcDs have a higher excitability and can elicit action potential firing which escapes somatic inhibition (Thome et al., 2014; Hodapp et al., 2022). Another example for AcDs being special dendrites comes from a recent study on chick tectal Shepherd's crook neurons (Weigel et al., 2022). These neurons typically possess an apical AcD which receives excitatory visual input whereas the basal dendrite receives auditory input. Synaptic activation of the apical dendrites was slower and evoked more prolonged currents due to a dendritic-specific contribution of NMDA receptors (Weigel et al., 2022). Although it remains to be proven, it is tempting to speculate that the AcDs of neocortical BCs share similar features. The privilege of short-circuit action potential generation appears to be beneficial for both AcD and BC axons. Branching and terminal density increase within and around the dendritic field of the parent AcD cell. This may help AcD axons to form synaptic connections more rapidly and deliver more inhibition to nearby pyramidal cells from which the parent BC receives most of its excitatory input.

The AcD and its axon may mutually support each other. One might have assumed that metabolic or material transport via the needle eye of the joint segment becomes limited but, at least in the time window analyzed, this apparently did not delay the growth of either neurite. BCs richly express calcium permeable AMPA receptors, excitatory synapses onto BC dendrites are strong and interneuronal dendritic tip elongation comes with the formation of new excitatory synapses (Chen et al., 2011). Possibly, action potential backpropagation might contribute to stabilize the larger dendrite. NMDA receptor signaling has been shown in developing mouse olfactory bulb mitral cells to stabilize one of the primary dendrites by suppressing RhoA, and concurrently this ‘winner’ dendrite triggers the pruning of the other dendrites (Fujimoto et al., 2023). Further, parvalbumin interneurons richly express tropomyosin receptor kinase B (TrkB) (Gorba and Wahle, 1999). The dendritogenetic effect of axonal TrkB receptor activation and axonal signaling endosomes has been demonstrated for cortical neurons (Moya-Alvarado et al., 2023). The AcD is at an ideal position for receiving a major share of such endosomal signals. However, there appear to be limits because the optogenetic stimulation was not able to increase the AcD local complexity further. By contrast, in BCs with somatic axons, a comparable axonal complexity has been evoked with the optogenetic stimulation. It remains to be shown whether these more complex BC axons elicit a stronger inhibition. As hypothesized (Gonda et al., 2023), a stronger inhibition may have contributed to the stunted growth of pyramidal cells in optogenetically stimulated OTC.

Cortical interneuron types may have distinct developmental periods during which they are most sensitive to activation or deprivation. Thus, the growth-promoting effect which we demonstrated to be specific for BCs might have been due to the analyzed time window, which overlaps best with BC differentiation. Axo-somatic innervation begins to form between P5 and P9, axo-somatic presynapses are well developed at P14 and mouse IPSC frequency increases from P6-P15 in mouse somatosensory cortex pyramidal cells, with most IPSC originating from perisomatic innervation (Kobayashi et al., 2008; Micheva et al., 2021). In line with this, exposure to the pro-inflammatory cytokine leukemia inhibitory factor (LIF) from DIV 12 onwards impairs the morphological and neurochemical differentiation of BCs, whereas non-BCs are barely affected (Engelhardt et al., 2018). Silencing the excitatory synaptic inputs in juvenile mice impairs dentate gyrus BC axon structure and granule cell inhibition due to subnormal NMDA receptor signaling in the BC (Pieraut et al., 2014; Feng et al., 2021). It remains to be shown whether the feature ‘complex AcD/complex axon’ persists through older ages or whether axons of somatic origin will catch up in dimensions given some more time. To this end, the results suggest that the AcD configuration offers a developmental advantage to BCs.

For the non-BCs, the lack of any axonal effect was surprising when considering that non-BCs have AcDs to a much higher proportion of up to 50%, and many axons emerge with substantial distance to the soma (Höfflin et al., 2017; Wahle et al., 2022). The dendrite-targeting axonal innervation of pyramidal cells develops earlier than axo-somatic innervation starting at P5 in mouse somatosensory cortex (Gour et al., 2021). Reducing non-BC excitability by expression of Kir2.1 channels from embryonic day (E) 15 in mouse cortex drastically decreases the average axonal length of calretinin and reelin neurons at P8 (De Marco Garcia et al., 2011). Possibly, the AcD of non-BCs has a growth promoting effect at younger ages. Yet, the non-BCs were responding to the DIV 11-15 optogenetic stimulation, albeit with a reduction of dendritic growth. Similarly, we recently reported that optogenetically stimulated pyramidal neurons analyzed from the same culture material develop shorter dendrites (Gonda et al., 2023). Interneurons have been shown to constantly remodel their distal dendrites (Chen et al., 2011). Such a dynamic might render them sensitive to activity-dependent regressive events. However, the AcD of the non-BCs was spared and it remained one of the longest dendrites, suggestive of a positive influence of the AcD configuration in non-BCs. To this end, we present the first evidence for a morphogenetic role of the AcD configuration.

## MATERIALS AND METHODS

### Ethics

Work was carried out in compliance with Ruhr University Bochum Animal Research Board and the Federal State of North Rhine-Westphalia.

### Culture preparation and transfection

Interneuronal axons and dendrites were reconstructed from the very same culture batches used for analysis of pyramidal cell dendrites (Gonda et al., 2023). Briefly, roller-type cultures were prepared from P1-P2 Long-Evans hooded rat visual cortex. Parasagittal slicing anticipated the tendency of large BCs to extend more in the anterior-posterior direction, as revealed by biocytin-filled BC axons reconstructed from stacks of 80 µm thick tangential sections of cat visual cortex covering fields of 2.3×2.2 and 3.8×1.7 mm^2^ with their radiating collaterals (Kisvárday et al., 1993). In slice cultures, the outgrowing main axonal arms will eventually adapt to the more 2D environment as roller cultures flatten over time. At DIV 8, cultures were transfected (Helios Gene Gun, Bio-Rad) with plasmids encoding CMV-driven hChR2(H134R)-eYFP (RRID: Addgene plasmid #20940).

### Optogenetic stimulation and selection of pulse frequency and duration

Cultures were maintained in a dark room to minimize exposure to white light. Stimulation was carried out using a custom-designed illumination setup equipped with 3×8 individually switchable 465 nm LED (Osram Oslon SSL80; Lumitronix), computer-controlled via an Arduino unit as recently described in detail (Gonda et al., 2023). The distance between cultures and LED was 12 mm. Light intensity determined with a photodiode (S130VC; Thorlabs) and a power meter (PM100D; Thorlabs) at the level of the cultures in the culture tubes was 0.7 mW/mm^2^. Cultures were exposed from DIV 11-15 to three rounds of blue LED stimulation at 0.5 Hz daily. Each round had 3×5 min stimulation with 5 min pauses during which the handling controls were mock-stimulated in the setup, a 30 min break, and again 3×5 min with 5 min pauses for mock-stimulating the controls; this way each round of stimulation lasted 1.5 h in total. The 0.5 Hz frequency has been effective in altering pyramidal cell dendritic growth (Gonda et al., 2023). Whole-cell illumination efficiently evokes neuronal spiking and action potential backpropagation (Grossman et al., 2010).

Previous work has shown that overexpression of specific glutamate receptor subunits increases dendritic complexity because the amplitude of depolarizing events increases with more receptors (Hamad et al., 2011; Jack et al., 2019). Event frequencies in our cultures around DIV 10 were ∼0.01 Hz and, for example, the overexpression of GluK2(Q) in pyramidal cells causes an increase of the frequency of calcium events to ∼0.06 Hz, which results in the growth of apical dendrites (Jack et al., 2019). Calcium events were at 0.02-0.04 Hz at DIV 11-15, and at DIV 18-20 individual pyramidal cells expressing genetically encoded calcium indicators display 0.1 Hz (Hamad et al., 2014; Jack et al., 2019; Engelhardt et al., 2018; Gasterstädt et al., 2022). The lowest frequency of 0.01 Hz matches the frequency of spontaneously occurring calcium events reported for supragranular neurons of the anesthetized mouse primary visual cortex at P8, and at P11 ∼0.03 Hz are reported (Rochefort et al., 2009). Calcium event frequency of ∼0.25 Hz occurs after eye opening and further increases to 0.5 Hz by the end of the fourth postnatal week (Rochefort et al., 2009), whereas the number of cells contributing to the events decreases substantially after eye opening *in vivo* (Rochefort et al., 2009). Together, this determined our choice of 0.05 Hz as ‘immature’ frequency, 0.5 Hz as frequency reached during the third postnatal week and 2.5 Hz as a frequency which is not naturally occurring during our window of analysis. In fact, the 0.5 Hz stimulation turned out to be effective, whereas the 0.05 Hz frequency did not alter dendritic growth and the 2.5 Hz evoked dendritic injury (Gonda et al., 2023).

Light pulse duration was 70 ms and 140 ms. In particular, the latter evoked a strong increase of c-Fos protein in ChR2-transfected neurons, and both durations were altering pyramidal cell dendritic growth. Therefore, as in previous studies (Gonda et al., 2023), we pooled the neurons of the 70 ms and 140 ms conditions for the axon analysis.

### Immunostaining

At DIV 15, ∼3 h after the final round of stimulation, cultures were fixed and immunostained with mouse anti-GFP antibody (1:1000; clone GSN24, G6795, Sigma-Aldrich, RRID: AB_563117) as described (Gonda et al., 2020; Gasterstädt et al., 2022). The batch-internal ‘handling’ control received the same ChR2-eYFP transfection and the same time in the illumination setup without any LED light, making the stimulation the only experimental variable. A handling control is essential because placing culture tubes in and out of the illumination setup, as gentle as it was done, could potentially generate mechanical stress which has been shown to contribute to dendritic remodeling (Franze et al., 2009).

### Inclusion and exclusion criteria for selection of interneuron types

BCs feature quite thick initial axons emerging from the soma or from a dendrite (Kisvárday et al., 1993; Höfflin et al., 2017; Wahle et al., 2022). The initial axon gives rise to major collaterals forming horizontal connections in L2/3 or in infragranular layers, respectively. Delicate collaterals emerge which quicky become varicose and form a dense local plexus within the dendritic field. Collaterals are studded with irregular-sized and partly large boutons and numerous short varicose axo-somatic terminal elements ending in close apposition to pyramidal-shaped somata and their proximal dendrites. Therefore, by inclusion criteria, our sample comprised the classical large horizontal BCs and smaller BCs with more local plexuses (Fig. 1; Fig. S1A1-A3).

As non-BCs we included cells with arcade-shaped axon arbors (Fig. 1B; Fig. S1B1-B3) and bitufted cells (Fig. 1; Fig. S1C1-C3), which were most frequently labeled in our material. Non-BCs are primarily dendrite-targeting cells with an adapting firing pattern (Markram et al., 2015; Feldmeyer et al., 2018). Their axons often emerge from dendrites (Höfflin et al., 2017; Wahle et al., 2022) and branch more sparsely than BC axons. Collaterals have rather small regular-sized boutons and project translaminar without extending into layer 1.

We excluded Martinotti cells for their highly variable axon length in layer 1. Somatostatin-positive Martinotti cell axons have been measured after genetic labeling in mouse cortex at P21. Total axonal length varies from ∼5 to >25 mm (Lim et al., 2018). Harvesting by chance a few Martinotti cells with extremely long or very short collaterals in layer 1 for either one of our experimental conditions could have easily led to false-positive results. Axons of translaminar somatostatin cells (types included in our study) display an average length of 25-30 mm at P21 and thus had a lower individual variability (Lim et al., 2018). Chandelier cells were not included due to immaturity, because axo-axonic innervation becomes recognizable by P14 and older (Pan-Vazquez et al., 2020; Gour et al., 2021; Micheva et al., 2021), beyond the time window analyzed here. We thus excluded layer 2/3 interneurons with prominent dendrites oriented towards layer 1, a typical feature of chandelier cells (Taniguchi et al., 2013). We also excluded bipolar cells because their small somata were too rarely transfected with biolistics and their axons are thin, sparsely branched and not always entirely labeled. Last, we excluded occasionally transfected neurogliaform neurons recognizable by their rather smooth axons of thin caliber and short dendrites (Staiger et al., 2015; Feldmeyer et al., 2018).

### Morphometry

Reconstructions derive from 86-slice cultures from 19 independent preparations, each carried out with five or six perinatal rats of both sexes, and slices from every animal were allocated to all experimental conditions run with each preparation. We reconstructed without preselection all neurons of the desired types which had completely stained dendrites and/or completely stained axonal plexuses. With sparse transfections, cells ideally resided in a solitary position allowing for 3D manual reconstruction (Neurolucida, MicroBrightField) carried out by two trained observers who were unaware of the condition. Reconstructions were analyzed with the Neurolucida software and the Neurolucida 360 Suite. In total, dendrites of 466 interneurons were reconstructed (*n*=65 cells at 0.05 Hz; 286 cells at 0.5 Hz; 115 cells at 2.5 Hz). For dendrites, we determined the mean dendritic length and segment number per cell for the three stimulus frequencies. Only the 0.5 Hz stimulation altered interneuronal dendritic complexity, and this was also the case for pyramidal neurons (Gonda et al., 2023). Therefore, we analyzed the 0.5 Hz sample separated by cell type (BC: 59 control cells, 69 stimulated cells; non-BC: 59 control cells, 99 stimulated cells). Because we did not see evidence for a distinct effect of stimulus duration, we pooled the reconstructed cells of the 70 ms and 140 ms conditions.

Further, from the 0.5 Hz sample, we reconstructed a total of 132 completely stained interneuronal axons (*n*=67 BC axons, 65 non-BC axons; reconstructed axonal length in total ∼3.2 meter). For axons, we determined total length, the number of branch points (nodes; all classified as bifurcations) per 1000 µm, bouton terminaux per 1000 µm, maximum branch order and the mean terminal segment length. Complexity of the axon and spatial distribution of the terminal endings were analyzed with soma-centered Sholl analysis and 1D linear axograms starting at the axon origin, which may be somatic or dendritic. The *z*-span of the axon in the OTC was determined after 90° rotation or the reconstruction and measuring the largest distance reached by collaterals. Somatic area was determined as proxy for soma size. The Sholl analysis of axonal endings was carried out with 50 µm bins. Next, we averaged the counts of two adjacent bins to work against variability (terminal endings are sometimes densely clustered). We eventually plotted the average, and the graphs thus report the average number of terminal endings of every second bin, plotted at 100 µm distance to the soma. This way the axes of the graphs were kept at the same dimension for easier comparison between the plots.

### Statistical analysis

Graphs and statistics were prepared using SigmaStat12.3 (Systat Software GmbH). Statistical comparison was carried out using non-parametric Mann–Whitney rank sum tests or ANOVA on ranks with Dunn's correction for multiple testing where appropriate. All data presented in the graphs are provided in the first data sheet of the Excel source data files in Tables S1, S2, S3, S4, S5; detailed information on statistical comparisons showing a significant difference is given in the second data sheet.

## Supplementary Material

10.1242/develop.202305_sup1 Supplementary information

Table S1. Dendrites of interneurons. Length [μm] and segment number of all interneuronal dendrites reconstructed from control and optogenetically stimulated cultures @0.05 Hz, 0.5 Hz, and 2.5 Hz. Length [μm] and segment number of BC and non-BC dendrites reconstructed from control and optogenetically stimulated cultures; 0.5 Hz condition, 70 ms and 140 ms pooled. Length [μm] and segment number of AcD of BC and AcD non-BC compared to regular dendrites of AcD cells and of somatic axon cells; 0.5 Hz condition, 70 ms and 140 ms pooled.

Table S2. Dimensions of BC and non-BC axons. Summary of general axon measures.

Table S3. Sholl-type analyses reveal denser arborizations of optogenetically stimulated BC, but not non-BC axons. Soma-centered Sholl analysis of control and stimulated BC and non-BC axons including total intersections. Axogram analysis of control and stimulated BC and non-BC axons including total intersections. Sholl analysis of terminal endings of control and stimulated BC and non-BC axons including total intersections.

Table S4. Axonal dimensions do not depend on culture z-span and soma size. Sholl circle intersections at 200 μm radius versus the z-span of the axonal plexus for BC and non-BC. Total axonal length versus z-span of the plexus, and versus somatic area of BC and non-BC. Axonal length analysis with regard to axonal origin.

Table S5. Sholl-type analyses reveal a higher local complexity of BC axons originating from a dendrite. Handling control axons separated by origin from soma or dendrite. The 0.5 Hz stimulated axons separated by origin from soma or dendrite. Handling control and stimulated axons originating from somata.
