# Supplementary material for: Axons of cortical basket cells originating from dendrites develop higher local complexity than axons emerging from basket cell somata
Source: Development. 2023 Nov 20;150(22):dev202305. doi: 10.1242/dev.202305 (PMC10690106; doi:10.1242/dev.202305)
Supplement: Supplementary information [file develop-150-202305-s1.pdf]

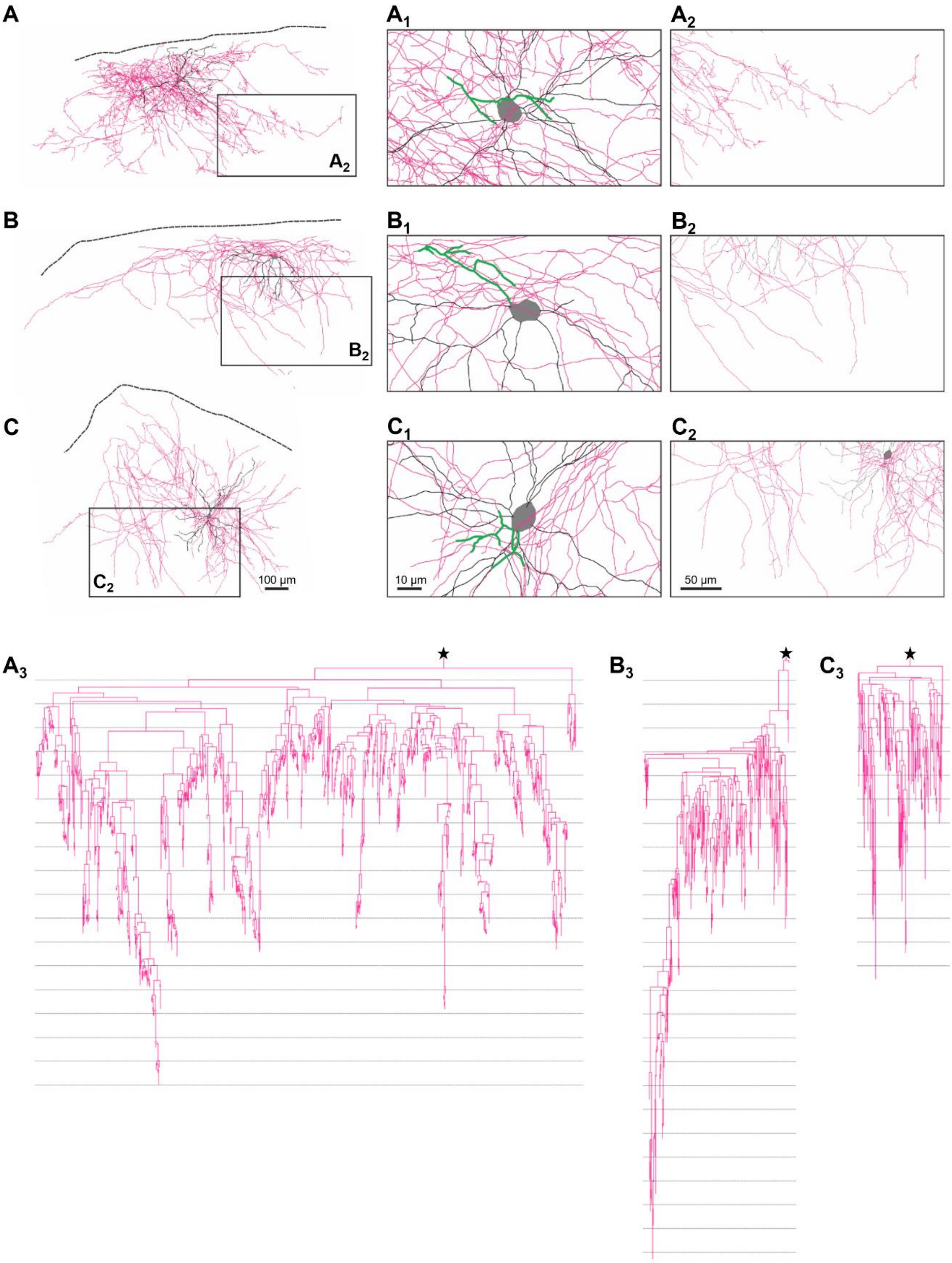

Development • Supplementary information

Fig. S1. Reconstructions of representative neurons. (**A, A1-A3**) Basket cell. (**B, B1-B3**) Arcade axon cell. (**C, C1-C3**) Bitufted cell. Left row, the total arborization. Dashed lines indicate the border of the slice (former pial surface). Soma in gray, dendrites in black, axons in light red. Middle row, a close-up of the arborization around the parent soma. The initial axon with its proximal collaterals is highlighted in greenish color. Right row, the region boxed in the reconstructions at higher magnification to show details of the terminal endings. The axograms (A3, B3, C3) with 100 µm bin width (the thin horizontal lines) are arranged below. The axon origin is marked by the asterisks. All axograms are at the same magnification. Note the many short endings supplied by the Basket axon collaterals (A3) versus the rather long terminal elements of the arcade (B3) and bitufted cell (C3) axon collaterals.

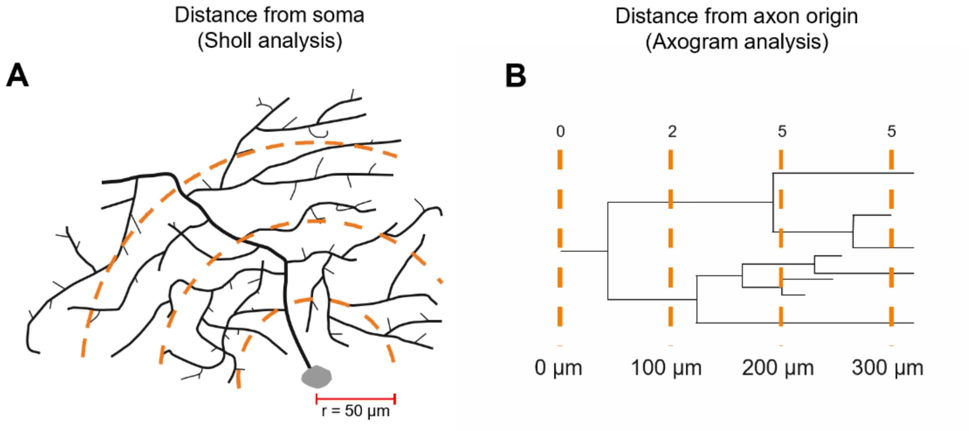

Fig. S2. (**A**) Principle of soma-centered Sholl analysis counting crossings of circle lines at 50 µm bins. (**B**) Principle of linear axogram analysis at 100 µm bins starting at the beginning of the axon (soma or dendrite). Line crossings were determined with the MicrobrightField NeuroExplorer and Neurolucida Suite 360.

Development • Supplementary information

Table S1. Dendrites of interneurons. Length [µm] and segment number of all interneuronal dendrites reconstructed from control and optogenetically stimulated cultures @0.05 Hz, 0.5 Hz, and 2.5 Hz. Length [µm] and segment number of BC and non-BC dendrites reconstructed from control and optogenetically stimulated cultures; 0.5 Hz condition, 70 ms and 140 ms pooled. Length [µm] and segment number of AcD of BC and AcD non-BC compared to regular dendrites of AcD cells and of somatic axon cells; 0.5 Hz condition, 70 ms and 140 ms pooled.

Available for download at
https://journals.biologists.com/dev/article-lookup/doi/10.1242/dev.202305#supplementary-data

Table S2. Dimensions of BC and non-BC axons. Summary of general axon measures.

Available for download at
https://journals.biologists.com/dev/article-lookup/doi/10.1242/dev.202305#supplementary-data

Table S3. Sholl-type analyses reveal denser arborizations of optogenetically stimulated BC, but not non-BC axons. Soma-centered Sholl analysis of control and stimulated BC and non-BC axons including total intersections. Axogram analysis of control and stimulated BC and non-BC axons including total intersections. Sholl analysis of terminal endings of control and stimulated BC and non-BC axons including total intersections.

Available for download at
https://journals.biologists.com/dev/article-lookup/doi/10.1242/dev.202305#supplementary-data

Table S4. Axonal dimensions do not depend on culture z-span and soma size. Sholl circle intersections at 200 µm radius versus the z-span of the axonal plexus for BC and non-BC. Total axonal length versus z-span of the plexus, and versus somatic area of BC and non-BC. Axonal length analysis with regard to axonal origin.

Available for download at
https://journals.biologists.com/dev/article-lookup/doi/10.1242/dev.202305#supplementary-data

Table S5. Sholl-type analyses reveal a higher local complexity of BC axons originating from a dendrite. Handling control axons separated by origin from soma or dendrite. The 0.5 Hz stimulated axons separated by origin from soma or dendrite. Handling control and stimulated axons originating from somata.

Available for download at
https://journals.biologists.com/dev/article-lookup/doi/10.1242/dev.202305#supplementary-data

Development • Supplementary information
