# Supplementary material for: Axons of cortical basket cells originating from dendrites develop higher local complexity than axons emerging from basket cell somata
Source: Development. 2023 Nov 20;150(22):dev202305. doi: 10.1242/dev.202305 (PMC10690106; doi:10.1242/dev.202305)
Supplement: Supplementary file 1 [file develop-150-202305_review_history.pdf]

Axons of cortical basket cells originating from dendrites develop higher local complexity than axons emerging from basket cell somata

Steffen Gonda, Christian Riedel, Andreas Reiner, Ina Köhler and Petra Wahle
DOI: 10.1242/dev.202305

Editor: Francois Guillemot

Review timeline
Original submission: 28 August 2023
Editorial decision: 5 October 2023
First revision received: 20 October 2023
Accepted: 24 October 2023

Original submission

First decision letter

MS ID#: DEVELOP/2023/202305

MS TITLE: Axons of cortical basket cells originating from dendrites develop higher local complexity than axons emerging from basket cell somata

AUTHORS: Steffen Gonda, Christian Riedel, Andreas Reiner, Ina Köhler, and Petra Wahle

I have received the reports of two referees on your manuscript and I have reached a decision. The reports are appended below and you can access them online: please go to and click on the 'Manuscripts with Decisions' queue in the Author Area.

The reviewers' overall evaluation is positive and we would like to publish a revised manuscript in Development, provided that you satisfactorily address the minor suggestions of the referees. Please attend to all these comments in your revised manuscript and detail them in your point-by-point response. If you do not agree with any of their criticisms or suggestions explain clearly why this is so.

Reviewer 1

Advance summary and potential significance to field

1. Further quantitative data are provided about features of axon bearing dendrites, in particular of basket cells in roller culture at several developmental stages.
2. Features are compared with equivalents for pyramidal neurons and between BC dendrites that do or do not carry axons, with greater complexity reported for axon-carrying dendrites. This is an important result with relevance to heterogeneous properties of dendrites within a single neuron and across different neurons of the same or different types.
3. Notably, the Authors compare the effect of spontaneous activity and optogenetic stimulation (at several pulse frequencies and durations), and conclude that spontaneous activity plays a greater role in the configuration of the axon arbor.

Comments for the author

© 2023. Published by The Company of Biologists under the terms of the Creative Commons Attribution License (https://creativecommons.org/licenses/by/4.0/).

I find the manuscript overall well-motivated and well-presented. Some minor suggestions:
1) Some of the background text in the Introduction might be condensed and then moved to the Discussion for further development.
2) The background material is very broad. This is appropriate, but I suggest it would be helpful to routinely specify the particular species and area.
3) It would be interesting to have a bit more discussion of axon carrying dendrites in different cell types, areas, and species. That is, do the Authors consider this an essential feature? Is the % somehow significant; ie, some neurons, but not all? Any implications for dendritic heterogeneity in general (i.e., for pyramidal neurons: basal, apical, and oblique dendrites?)
4) I found the pink shading somewhat distracting. The Authors might consider shifting the color scheme: black for the preponderant axon arborization and pink for the soma and dendrites.

Reviewer 2

Advance summary and potential significance to field

In this paper, Gonda and collaborators focussed on axons originating from dendrites in either basket cell (BC) GABAergic interneurons or in other GABAergic interneurons (non-BC). They showed that axons originating from BC dendrites have distinct morphological properties compared to axons originating from BC somata.
Further, they showed that optogenetic stimulations during a short sensitive period affects the complexity of soma-originating but not dendritic originating axons. This study is a quantitative tour de force, including data from many BC and non-BC (from organotypic cultures). The introduction and discussion are exhaustive, and the data carefully presented and interpreted. While the study does not provide mechanistically advances on interneurons axon morphogenesis, it constitutes an important information database on which future study can be based.

Comments for the author

I have very minor suggestions, listed below.

_ While the number of BC and non-BC analyses can be found in the legend and the number of organotytpic cultures can be found in the methods, it would help the reader if this Infos could be grouped int he same place, so the reader can immediately understand how many BC (or non-BC) have been analyzed from how many cultures and the average neurones reconstructed per cultures.

_ The proportion of axons originating from BC somata vs axons originating from BC dendrites could be stated early on in the results section. Where BC with dendrite-originating axons randomly detected in different cultures or where they clustered, for example with multiple BC with dendrite-originating axons detected in the same cultures?

_ the title of the legends is not very informative. Stating a take one message for each figures would help the reader

---

First revision

Author response to reviewers' comments

MS ID#: DEVELOP/2023/202305

MS TITLE: Axons of cortical basket cells originating from dendrites develop higher local complexity than axons emerging from basket cell somata.

AUTHORS: Steffen Gonda, Christian Riedel, Andreas Reiner, Ina Köhler, and Petra Wahle

© 2023. Published by The Company of Biologists under the terms of the Creative Commons Attribution License (https://creativecommons.org/licenses/by/4.0/).

Dear Dr. Guillemot,
we have been very happy to read these supportive reviews and that the referees appreciate our „quantitative tour de force".

We have now revised the manuscript following your instructions. In fact we like to supply all source data, although in many papers that I checked, the table works underlying the graphics are not included. We left them in (Excel files to be linked in the Supplementary pdf), including the statistical tests, and hope that this will be appreciated.

All Supplementary Tables (our source data) have been quoted in the text, together with the graphical Figures to which they belong.

All items have now been uploaded as separate files.

The length of Intro, Results, Discussion and Figures legend is below 6.000 words. The Methods section has 1.700 words. The Abstract is shorter than 180 words, we removed the paragraph „Teaser".

Reference list and Authors Contributions are Journal style. The small changes done are marked in red text in the „track changed marked" version. A clean copy is also provided.

Specific changes as requested by the referees are as follows.

Referee No. 1:

Point 1. We left the background information in the Introduction. In our view, the general aspects of structural plasticity are better in such a compact form. Also, we preferred to leave the Discussion section in its present form rather than overloading it.

Point 2. We added the requested information on species (mostly rodent) and brain areas.

Point 3. We feel that the referee's questions on axon-carrying dendrites are better suited for a review article. In fact, recent reviews have summarized what is currently known, and we also discussed several issues in our recent paper (Wahle et al., eLife, 2022), for instance that in pyramidal cells the basal, not the apical, dendrites carry the axon. To this end, yes, we regard the AcD configuration as an essential feature, and the novel data on axon and dendritic growth are the first to report functional implications during development.

Point 4. We changed the color of the axons in Figure 1, avoiding the pink.

Referee No. 2:

We thank the referee for appreciating the „tour de force". We agree, mechanisms need to be unraveled. We did at least discuss some very likely mechanisms, and believe that the well characterized mechanisms of neurite growth will also be seen for this particular morphological configuration.

Point 1. We compiled the information on cell numbers and number of OTC now into the one paragraph in the Methods section.

Point 2. This is an important point. It opens an avenue of speculations. First, we do not have the impression that this feature occurs in a clustered manner. To this moment, it seems random. A given OTC can have 1, or 2, or no, AcD cell. The more transfectants, the higher the chance to find an AcD interneuron. All proportions mentioned are derived from counts of mammalian cortex *in vivo* (Wahle et al., eLife 2022).

The random occurrence seems also the reason why we had to prepare and check so many cultures in order to collect the necessary numbers of AcD interneurons. An (admittedly weak) argument supporting the impression „random" is that we obtained very similar proportions of pyramidal cells

© 2023. Published by The Company of Biologists under the terms of the Creative Commons Attribution License (https://creativecommons.org/licenses/by/4.0/).

with AcD in all mammalian species and all cortical areas (extensive for the monkey brain; Wahle et al., eLife 2022). Intriguingly, however, individuals within the species varied in case of pyramidal AcD cells. Whether this will hold for interneurons, though, is a matter of future research which should best be resolved *in vivo*. To speculate a little bit (of course not included in the revised manuscript): lets image that individual immature human beings happen to have more AcD Basket cells maturing more rapidly delivering more and stronger local inhibition, while other individuals have less. Could that potentially impact the temporal and spatial development of cortical network activity and, eventually, cognitive performance of the individual?

To this end we included one sentence in Results section stating „We did not have the impression that AcD interneurons occur clustered. Any given OTC can have 1, or 2, or no AcD interneuron, and the more transfectants the higher the chance to find an AcD interneuron."

Point 3. The titles of the graphical Figure Legends are now „active" titles to give a take-home message.

We hope that the changes are in agreement with the suggestions of the referees.

On behalf of all co-authors

Petra Wahle

Second decision letter

MS ID#: DEVELOP/2023/202305

MS TITLE: Axons of cortical basket cells originating from dendrites develop higher local complexity than axons emerging from basket cell somata

AUTHORS: Steffen Gonda, Christian Riedel, Andreas Reiner, Ina Köhler, and Petra Wahle
ARTICLE TYPE: Research Article

I am delighted to tell you that your manuscript has been accepted for publication in Development, pending our standard ethics checks.

© 2023. Published by The Company of Biologists under the terms of the Creative Commons Attribution License (https://creativecommons.org/licenses/by/4.0/).
